# Effect of neuromuscular taping on musculoskeletal disorders secondary to the use of aromatase inhibitors in breast cancer survivors: a pragmatic randomised clinical trial

**DOI:** 10.1186/s12906-018-2236-3

**Published:** 2018-06-11

**Authors:** Inmaculada Conejo, Bella Pajares, Emilio Alba, Antonio Ignacio Cuesta-Vargas

**Affiliations:** 1 Department of Physiotherapy, Faculty of Health Science, University of Málaga, Málaga, Spain; 2 Department of Medical Oncology, Carlos Haya Regional University Hospital, IBIMA, Málaga, Spain; 3 Department of Physiotherapy, IBIMA, University of Málaga, Málaga, Spain; 4 School of Clinical Science, Faculty of Health, Queensland University of Technology, Kelvin Grove, Australia

**Keywords:** Breast cancer, Hormonal therapy, Aromatase inhibitors, Estrogen deprivation, Myalgia, Arthralgia, Carpal tunnel syndrome, Visual analogue scale, Pressure pain threshold, Neuromuscular taping, Kinesio taping

## Abstract

**Background:**

Aromatase inhibitors reduce breast cancer recurrence rates in postmenopausal women by about 30% compared with tamoxifen while treatments differ. Unfortunately, nearly half of women taking AIs report AI-associated arthralgia (AIA), leading to therapy abandon in on third of patients, which could lead to cancer recurrence. The purpose of the current study was to evaluate the effectiveness of Neuromuscular Taping (NMT) in the treatment of AIA in women who have been treated of BC.

**Methods:**

This study included 40 BC survivors receiving endocrine therapy (either AIs or TMX) from Hospital Universitario Virgen de la Victoria (Málaga, Spain) suffered from AIA. Patients were randomized to one of the two groups that made this pilot study: A. Placebo intervention B. Real NMT. Clinical data were collected from medical history, grip strength, algometry measured, questionnaires and VAS scale. There have been three interventions prior to the completion of the study, 5 weeks later. The primary objective of this pilot study was to achieve an improvement of pain by 20% decrease of VAS.

**Results:**

Significant differences in measures of VAS (*p* = 0.009), global health status/QoL (*p* = 0.005), fatigue (*p* = 0.01) and pain (*p* = 0.04) were observed post intervention with NMT.

**Conclusions:**

An intervention by NMT to MSCM under treatment with AIs improves their subjective sensation of pain. In addition, this taping had an impact on variables related to the quality of life. This pilot study may be the basis for others to support the use of NMT for the treatment of AIAs, thereby improving their well-being and reducing the dropout rate.

**Trial registration:**

ClinicalTrials.gov Identifier: NCT02406794. Registered on 2 April 2015 Retrospectively registered.

## Background

Breast cancer (BC) is the most common cancer in women and the leading cause of death among middle-aged women in developed countries. The latest available estimation, for 2012, is that there would be 1.671.149 new cases worldwide and 521.907 deaths [1]. In Spain, the most frequently diagnosed tumor is BC (25,215 new cases), mortality was only of 6.075 patients, so its prevalence at 5 years is 104.210 [2]. Both factors, the increase in the number of new cases and lower mortality, due to recent scientific advances in screening, diagnosis and treatment, result in a higher prevalence of BC and an increase in the number of breast cancer survivors (BCS) [3].

Aromatase inhibitors (AIs) interrupt the conversion of androgens to estrogens in postmenopausal women, thus able to reduce circulating estrogen levels to about 1/10th of normal levels. Currently, the most widely used in clinical practice are the third generation aromatase inhibitors (exemestane, anastrozole and letrozole). AIs improve outcomes in the adjuvant treatment of postmenopausal women with hormone receptor positive (HR+) BC compared with tamoxifen decreasing the risk of recurrence (20–36%) and decreasing 10-year breast cancer mortality (12.1% vs. 14.2% RR 0·85, 0·75–0·96; 2p = 0·009) [4].

TMX induced more gynecological and thromboembolic side effects, however patients receiving AIs have a higher incidence of osteoporosis, bone fractures and musculoskeletal symptoms, particularly pain and stiffness in the joints [5]. These co-morbidities associated with AIs compromises survivors’ quality of life and leads to non-compliance [6].

Is important to consider that to achieve the same benefits of adjuvant endocrine therapy in clinical practice as those observed in clinical trial settings [7], long-term compliance with treatment is required [8].

Adjuvant endocrine therapy is associated with poor compliance rates compared with chemotherapy and radiotherapy. Retrospective analyses indicate prescription-controlled adherence rates of 80% for tamoxifen and 69% for anastrozole [9].

The worst figures are found in those survivors who underwent both types of hormone therapy (from one form of therapy to the other), with dropout rates of 31.3% [10]. Induced AIs musculoskeletal toxicity may be the cause of this lower persistence rate and possibly reduced efficacy.

To analyze the problem of musculoskeletal disorders associated with the use of IAs, it is important to begin by correctly define these disorders, and thus have objective criteria for diagnosis. In general, these usually have joint pain that is localized mainly in the wrists, hands and knees symmetrically [5]. The Intergroup Exemestane Study (IES) performed retrospective studies which concluded that the occurrence of carpal tunnel syndrome (CTS) cases was higher in those patients who were treated with exemestane (2,8%) than in those who taking TMX as hormonal therapy (0,6%). The 69% of cases that manifest this syndrome, underwent surgical release, underwent surgical release [11]. The ATAC (Arimidex and Tamoxifen, alone or in combination) trial analysed the natural history of patients who presented with CTS during adjuvant treatment for breast cancer. CTS were reported in 2,6% of the participant in the anastrozole arm, compared with 0.7% in the tamoxifen arm, although the symptoms were mild or moderate in intensity [12]. Other symptoms such as morning stiffness, myalgia, tenosinovial changes and decreased grip strength were also found [13]. Occurrence of carpal tunnel syndrome and any musculoskeletal events can serve as biomarkers of treatment effectiveness, because the estrogenic reduction is a mechanism of action for both, antitumor effect and development of endocrine treatment-induced symptoms. So much so that musculoskeletal symptoms, including CTS, was associated with improved disease-free survival [11]. A recent meta-analysis found no difference in anti-tumor efficacy between the three AIs [14], and for this reason it would be interesting to know the side effects caused by each of them with prescriptive purposes.

Despite high prevalence of the arthralgia, which often accompanies the AIs medicine, the way this compromises their quality of life and leads to non-compliance; very little is known about what causes this difficult problem. Clearly, it is important informing the patient before therapy that joint pain is a very common side effect, and thus better tolerate the problem. Once symptoms debuting have been used therapies such as acupuncture to combat them [15]. In clinical practice are often used nonsteroidal anti-inflammatory drugs (NSAIDs) for improving musculoskeletal disorders and even get some relief, they are not exempt from a number of side effects. There are some particularly interesting NSAIDs (COX-2 selective inhibitor), but are known to alter the regulation of aromatase [16], and have potentially dangerous side effects, which means they are not recommended for routine treatment of AIs effects secondary. The ATAC trial showed that obese women (body mass index [BMI] > 30 kg/m2) had more joint symptoms than women with a BMI of 25–30 kg / m2 or those with a BMI < 25 kg/m2 [17], so another excellent intervention for these women would be weight loss. Some studies have shown the effectiveness of an intervention based on the practice of yoga in reducing musculoskeletal symptoms such as general pain, muscle pain and overall physical discomfort [18]. Other study focused on changes in rheumatological symptoms, such as morning stiffness, joint pain and grip strength and they found that a short course of low-dose prednisone showed promising potential as an effective treatment [19]. In a retrospective study about the usefulness of diuretics and bisphosphonates for managing arthralgia produced by hormone therapy, the study concluded that these drugs reduced the discomfort caused by the joint pains [20]. A recent meta-analysis published by Yang et al. reported that pharmacological approaches, acupuncture, and relaxation techniques showed moderate to large effects on pain, whereas nutritional supplementation and physical exercise had no significant effects on it [21]. It can therefore be concluded that there is no established, effective treatment for this difficult problem which affects up to half of women on AI therapy [6].

Neuromuscular Taping (NMT) has become an increasingly popular technique in recent years [22] and is mainly used in various areas of sports performance and/or rehabilitation. Kinesiology taping with elastic tape is a new therapeutic method indicated for the relief of pain, neuromuscular rehabilitation, musculoskeletal upheavals and related to sports injuries. The elastic characteristics of Kinesiology tape allow extending from a minimum of 120% to a maximum of 170% of its original length, after which recedes again at its original length [23]. The Neuromuscular Taping has achieved excellent results in decreased pain, increased functional abilities and in reducing edema [24]. This new technique of taping has demonstrated efficacy in the acute treatment of sports lesions [25], muscle disorders [26], has achieved improvement of balance and functional activities in neurological patients [27, 28], achieve better sensorimotor synchronization [29] reducing swelling and postoperative pain [30] and reducing lymphedema as a additional measure to the Complex Decongestive Therapy [31], although the edema reduction of multilayered bandages is better [32]. Nevertheless, no studies have researched its use for the treatment of musculoskeletal disorders in BCS, and its impact on the pressure pain threshold (PPT) or on VAS. The purpose of this study was assessing the effect of an intervention by NMT in the subjective sensation of pain in these patients.

## Methods

### Design and participants

The present study is a randomized controlled clinical trial conforming to Consolidated Standards of Reporting Trials (CONSORT) guidelines. This pragmatic randomized study was carried out with a sample of 40 patients who had been treated from BC with third generation AIs, and came to consultation with her oncologist at the University Hospital Virgen de la Victoria (Málaga, Spain). Patients were recruited from May 2016 to September 2016, two days each week, through convenience sampling. All patients gave their informed consent for the study. The trial had ethical approval of the Ethics Committee of the Provincial Investigation Malaga (Ministry of Health Andalusian Health Service, Spain). They were respected the principles of the Declaration of Helsinki. Inclusion criteria were: (a) higher 18 years old; (b) have suffered from primary breast cancer confirmed histologically (I-IIIA); (c) have completed primary carcinoma treatment (surgery, chemotherapy, radiotherapy); (d) be subjected to hormonal therapy as adjuvant therapy to the process by AIs (exemestane, anastrozole, letrozole); (e) present a functional status according to WHO from 0 (asymptomatic, complete and ambulatory activity) or 1 (symptomatic but completely ambulatory, strenuous physical activity restricted but able to perform sedentary gentle activities); (f) correctly understand Spanish; (g) show their approval by signing the informed consent; (h) participants must have submitted attributable to AIs musculoskeletal disorders (i) the painkillers were prescibed to the patients following the usual care from guidelines in both arms.

### Intervention

For this trial we have used data obtained between May 2016 and September 2016. All recruited participants were surveyed by the same trained interviewer, completing a data collection sheet about various sociodemographic and anthropometric variables. Partients as well filled self-report questionnaires about their mental state, their profile of mood state (POMS), fatigue evaluation (Quickpiper) and quality of life (EORTC QLQ-C30). After completion, we used a pressure algometer to assess their PPT and an analog dynamometer to measure grip strength of both hands. Then we used the VAS scale to evaluate their pain and three functional questionnaires were filled: Spine Functional Index (SFI), Upper Limb Functional Index (ULFI) and Backache Disability Index (BADIX). After that, participants went to nursing consultation, where it was extracted a blood sample. Each participant was then randomly assigned to one of the two groups that made this pilot study. Group A-Intervention Group: Real NMT over the areas in which they manifested pain. Group B-Placebo group: Sham NMT in the painful areas without any therapeutic technique.

The user received an envelope which contained a number, one or two, to determine which group would be part. The first group (A) received a decalogue of health advice (general, to lead an active lifestyle), drawn from the best available evidence, and was applied several strips of neuromuscular taping over the areas in which they manifested pain (cervical, lumbosacral, both or wrist-forearm). The physiotherapist who performed the tapping was always the same, had the proper certification for application and counted on 10 years of experience in this technique. The material used for this trial (Cure Tape®, Fysiotape BV Netherlands. Medical Device Class A, registration number NL/CA01/04–07434) is waterproof, breathable and adhesive. Its composition is latex-free and has elasticity of 130–140%. 5 cm wide tape. The color blue was used (0.51 mm thickness and density 440.56 kg/m3).

**Fig. 1:**
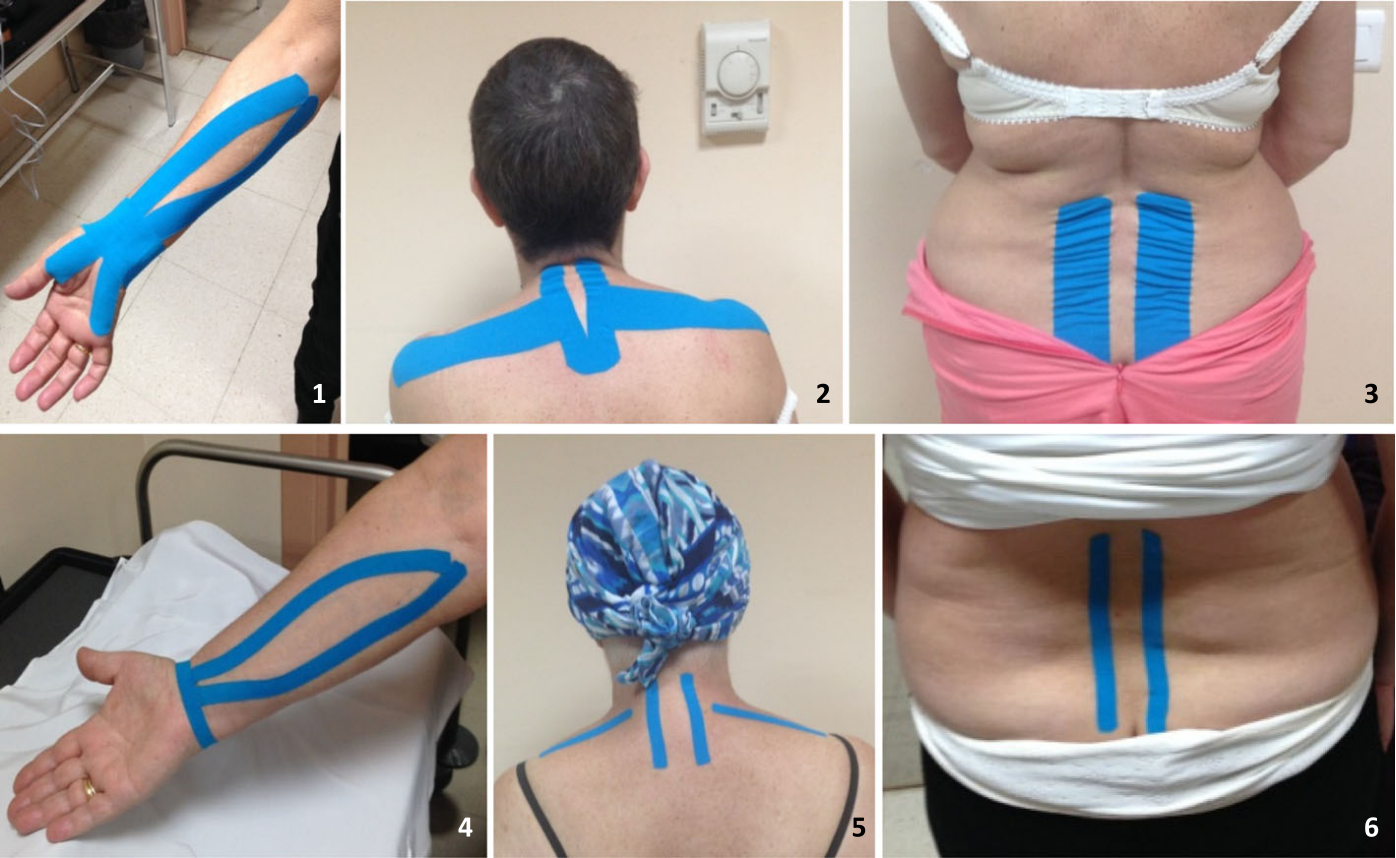
Different applications of NMT; 1, Application for carpal tunnel syndrome. 2, Application for cervical pain. 3, Application for lower back pain. 4, Sham application for carpal tunnel syndrome. 5, Sham application for cervical pain. 6, Sham application for lower back pain

Depending on the participant symptoms, the taping were placed on four possible locations (Fig. 1):

1. Carpal Tunnel.
2. Lumbar and cervical if morning stiffness.
3. Lumbar bandage.
4. Cervical bandage.

When the participant presented CTS diagnosed properly, a possible application involves placing two strips. The first helps relax the long palmar muscle using a 5 cms. Wide strip cut into an “X” shape. The crossing point was applied in neutral position on the palmar wrist area. The short anchors were placed on the little finger hypothenar region and thenar eminence of the thumb; we ask the participant to perform elbow extension, forearm supination and hand dorsiflexion, and then apply the functional strips surrounding the entire muscle belly, to the medial epicondyle of the humerus. The second strip is placed with a technique to increase the space in the wrist, for which we use a strip of 5 cms. Wide cut into an “I” shape. During laying on wrist, this is held in neutral position. The base is applied without tension from the front side of the radius, on the back of the wrist toward the front side of the ulna. Dorsally, a 50% stretch of was used to apply. There will be a gap between the base and the anchor. There will be a clearance between the base and the anchor [33].

In the case of localized lower back pain, this was the TNM technique used: two blue 5 cms. Wide strips cut into an “I” shape, without traction were applied from the origin of the lumbar erector spinae (iliocostalislumborum) to its insertion, to relax it. The base of the strip was applied to the sacral region (at the S1) in the neutral position. Then the participant was asked to take the spine into flexion and for each functional strip with slight lateral flexion; the tape was then used on one side paravertebrally on muscle belly in the direction of the cranium. The same procedure was then applied to the other side [34, 35].

In cases where the pain was located in the cervical area, the participant received a standardized therapeutic TNM application [36]. The first strip was a blue Y-strip (a 5 cms. Wide base strip cut into two 2,5 cms. tails) placed over the posterior cervical extensor muscles and applied from the insertion to origin without tension. The participant was placed in a sitting position and each tail of the strip was applied with her neck in a position of cervical flexion and heterolateral slight rotation. The laying direction was from the first dorsal vertebrae (T1-T2) toward cranial, to the upper-cervical region. Then we use a technique to relax the supraspinatus muscle. For this we utilized two 5 cms. Wide strips cut into an “I” shape that were applied without tension from the head of the humerus to the medial border of the scapula. For placement of functional strips we ask the participant to take the arm into adduction, and scapular girdle in detraction [33, 37]. Participants were explained that they should keep the application for 7 days.

The second group (B) was also given the decalogue of healthy tips and were applied a sham NMT that were placed in the painful areas without any therapeutic technique. The material and color of sham strips was the same, but these were more narrow (1.25 cms. wide) and shorter. They also were placed with the patient in neutral position, without utilizing muscle or increase space tecchnique used in the experimental group. After the taping intervention, we register again algometry, VAS and dynamometry. Both groups were called again to repeat all evaluations and reapply tape to 7 days. This process of data collection was carried out again at 5 weeks of the start of the study, along with the second and final blood extraction.

The purpose of this intervention was to improve participants pain, through the reduction by 20% of the values of their visual analogue scale (VAS) at various points, to relieve symptoms and reduce AIs therapy dropouts.

### Measurements

Medical and demographic information brought us information about their age, marital status, education level, anthropometric data, surgery and complementary treatments for BC. All these variables will be collected only once, on the first visit, prior to randomization. Plasma proteins.

Plasma proteins associated with arthralgia, cancer–related fatigue and insomnia [38], such a C-reactive protein (CRP) and creatine kinase (CK) were analysed by proteomic analysis. To perform this analysis was used a surface-enhanced laser desorption/ionization, a mass spectrometry (MS) technique, followed by further sample processing using one-dimensional gels and trypsin digest for protein identification using liquid chromatography and database searching [39].

Pain threshold pressure (PPT) is described as the minimum amount of pressure with which an initial feeling of pressure switches to pain [40].

Dynamometer.This strength evaluation has been previously used to measure the loss of strength in BCS [41], including cases where patients are undergoing hormone therapy with AIs [13].

### Patient-report outcomes

Profile of Mood States (POMS). This questionnaire completed by patients is used frequently for the study of BCS [42].

QuickPIPER. Questionnaire utilized to determine the degree of fatigue and it phenotype. The test-retest reliability of this tool is very good (*r* = 0.947, *P* < 0.001) [43].

EORTC QLQ-C30. It is a specific questionnaire to assess quality of life (QoL) of patients diagnosed with cancer. In some trials, QoL was further defined by specific impacts such as physical functioning. Also it serves to evaluate symptoms (most often pain) [44].

Visual Analogue Scale (VAS). It is a tool that was designed in order to allow a subjective assessment of pain. Previous studies have shown that this scale has adequate psychometric properties [45].

Spine functional Index (SFI-Sp). In this pilot study we have used the Spanish version (SFI-Sp), because it has proven to be a valid and reliable measure of the spinal region result [46].

Upper Limbs Functioal Index (ULFI-Sp). This trial used the Spanish version (ULFI-Sp). This quiz has proven useful for evaluating patients with disorders of the upper extremities [43].

Backache Disability Index (BADIX). This index contains a score of five movements of the trunk in an upright position that translate into back pain index (BAI) and a register of “morning stiffness in the back” (MBS). Their sum gives the BADIX [47] and appears to be a valid and reliable tool for the evaluation of morning stiffness [48].

### Sample size

Version 3.1 G Power was used to estimate sample size. A minimum of 78 subjects per group will be needed “a priori” to have sufficient statistical power (80%), alpha error (0.05) and size 0.4 effect on the visual analog pain scale [49].

### Statistical treatment

For analysis of the results it was developed a database using information gathered from participants notebooks, algometers, dynamometers and self-administered questionnaires (QuickPIPER, POMS, quality of life, VAS, SFI, ULFI and BADIX). Once intervention phase, descriptive statistics was performed with measures of central tendency and dispersion of the study variables. After this became an inferential analysis between the outcome variables in both groups. The means and a one-way analysis of variance (ANOVA) was used to asses differences between groups as well as the *p* value. The mean differences between the self-reported questionnaires as part of the intra-group analysis were examined. Finally t student and asymptotic significance was used to investigate the relationships between these variables at different study times. The means and a one-way analysis of variance (ANOVA) was used to asses differences between groups as well as the p value. The mean differences between the self-reported questionnaires as part of the intra-group analysis were examined. Finally t student and asymptotic significance was used to investigate the relationships between these variables at different study times. SPSS version 15.0 V for Windows.7 was used for data analysis.

## Results

### Demographic and clinical data (Fig. 2)

A total of 40 postmenopausal BCS, with a mean age of 66,30 years (50–82), enrolled and underwent baseline evaluation between May and September 2016. Both, the experimental and control groups had 20 participants. Women were married (47,5%), had primary/secondary level education (40%) and only 22,5% were employed. If we look at the data that inform us whether the body mass had a healthy value observed that 75% were overweight, mean waist circumference was 97,63 cms. and the hip, 110.19 cms. Most women had stage II BC (40%), and received both radiation and chemoterapy (47,5%) as adyuvant treatment after surgery. Respect to hormone therapy, 9 (22,5%) women had taken Tamoxifen prior to AIs, whereas the remaining 31 (77,5%) had only taken AIs. Additionally, the most common AI was Letrozol - Femara ®, 57,5% of the participants were taken it and the mean duration of treatment at the time of starting the intervention 17.40 months. None patient received physiotherapy treatment. If we value comorbidities present at the time the study began, besides the pain at different levels ((cervical (82.5%), shoulder (85%), lumbar (75%)and hands/wrist (62.5%)) the most frequent were hypertension (60%) and vascular disorders (57.5%). The ANOVA revealed that groups were not significantly different on age, those related to the assessment of overweight and obesity (weight, height, body mass index, waist perimeter and hip perimeter) and tamoxifen and AIs treatment duration. Table 1 summarises the patients demographic and clinical data. We compared the means of all outcome variables in the baseline and there were no significant differences in any of them, except BAI (*p* = 0.05), although both groups are not as similar as they were their descriptive characteristics, possibly due to the small sample size. As can be observed in Table 2, the groups were similar and comparable at the outset.

**Fig. 2:**
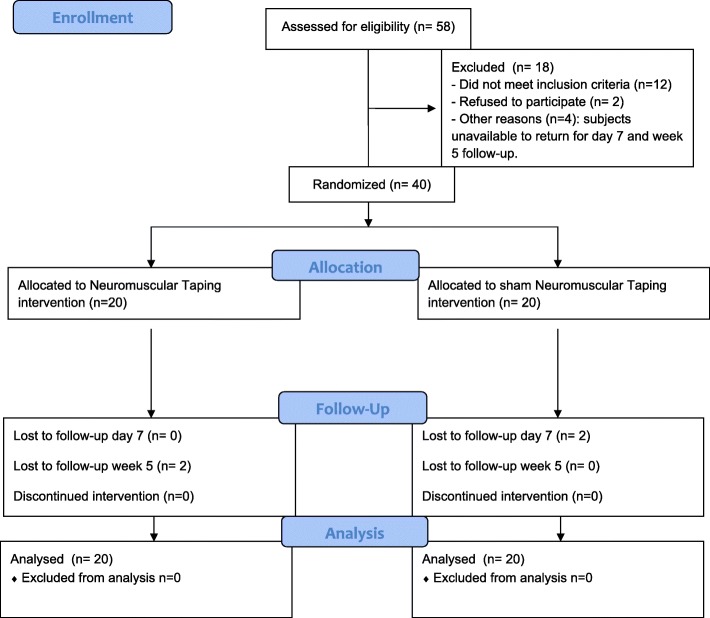
Participants Flow Diagram

**Table 1:** Mean or number (%). Range (Min-Max) and ANOVA of demographic and clinical baseline characteristics of groups

Characteristic		Groups		
	Total (*n* = 40)	Experimental (*n* = 20)	Control (*n* = 20)	F *(p)*
Age (yr). mean (Range)	66.30 (50–82)	67.80 (50–82)	64.80 (54–81)	0.95 *(0.33)*
Marital Status. number (%)				
Single	5 (12.5)	3 (15)	2 (10)	
Married	19 (47.5)	14 (70)	5 (25)	
Divorced/Separated	6 (15)	0 (0)	6 (30)	
Widow	10 (25)	3 (15)	7 (35)	
Education level. Number (%)				
Any study	11 (27.5)	7 (35)	4 (20)	
Primary/ Secondary School	16 (40)	7 (35)	9 (45)	
Vocational training/ Qualifications	8 (20)	3 (15)	5 (25)	
University/ Superior education	5 (12.5)	3 (15)	2 (10)	
Employed. number (%)	9 (22.5)	3 (15)	6 (30)	
Weight (Kg). mean. (Range)*	72.98 (44–130)	72.71 (53.2–97)	73.25 (44–130)	0.01 *(0.91)*
Height (cm). mean. (Range)*	159.60 (147–171)	159.25 (148–170)	159.95 (147–171)	0.11 *(0.74)*
Body Mass Index (BMI). mean. (Range)	28.17 (16.17–47.79)	28.80 (20.70–39.91)	28.62 (16.17–47.79)	0.01 *(0.92)*
Overweight (BMI ≥ 25). number (%)	30 (75)	14 (70)	16 (80)	
Waist perimeter (cm.). mean. (Range)	97.63 (68–147)	95.80 (75–120)	99.45 (68–147)	0.69 *(0.41)*
Hip perimeter (cm). mean. (Range)	110.19 (89–145)	111.85 (93–130)	108.53 (89–145)	0.72 *(0.40)*
Tumor grade. Number (%)				
Grade I	8 (20)	6 (30)	2 (10)	
Grade II	16 (40)	9 (45)	7 (35)	
Grade III	14 (35)	5 (25)	9 (45)	
Grade IV	2 (5)	0 (0)	2 (10)	
Kind of treatments. Number (%)				
Surgery (S)	3 (7.5)	3 (15)	0 (0)	
S+ Chemotherapy (ChT)	3 (7.5)	0 (0)	3 (15)	
S+ Radiotherapy (RT)	15 (37.5)	10 (50)	5 (25)	
S+ ChT+ RT	19 (47.5)	7 (35)	12 (60)	
Previous Tamoxifen (TMX). number (%)	9 (22.5)	3 (15)	6 (30)	
TMX treatment duration (mo). mean. (Range)	4.46 (0–32)	4.30 (0–32)	4.64 (0–25)	0.01 *(0.91)*
Aromatase Inhibitors (AIs). number (%)				
Anastrozole (Arimidex®)	12 (30)	4 (20)	8 (40)	
Letrozole (Femara®)	23 (57.5)	14 (70)	9 (45)	
Exemestane (Aromasin®)	5 (12.5)	2 (10)	3 (15)	
AIs treatment duration (mo). mean. (Range)	17.40 (2–52)	15.25 (2–52)	19.55 (2–44)	1.25 *(0.27)*
Comorbidities. number (%)				
Cervical pain	33 (82.5)	19 (95)	14 (70)	
Shoulder pain	34 (85)	18 (90)	16 (80)	
Lumbar pain	30 (75)	17 (85)	13 (65)	
Hands/ Wrist pain	25 (62.5)	12 (60)	13 (65)	
Hypertension	24 (60)	14 (70)	10 (50)	
Heart disease	8 (20)	3 (15)	5 (25)	
Circulation problems	23 (57.5)	12 (60)	11 (55)	
Thyroid problems	6 (15)	4 (20)	2 (10)	
Stroke	0 (0)	0 (0)	0 (0)	
Diabetes	5 (12.5)	2 (10)	3 (15)	
Gastric/ duodenal ulcer	1 (2.5)	0 (0)	1 (5)	
Breathing problems	6 (15)	2 (10)	4 (20)	
Parkinson	0 (0)	0 (0)	0 (0)	
Cognitive impairment	0 (0)	0 (0)	0 (0)	
Migraine/chronic headache	7 (17.5)	4 (20)	3 (15)	
Vision problems	13 (32.5)	7 (35)	6 (30)	
Hearing problems	10 (25)	6 (30)	4 (20)	
Lymphedema	4 (10)	2 (10)	2 (10)	

*Self-reported weight and height

**Table 2:** Mean (CI 95%) and analysis of variance (ANOVA) of baseline, Patient-Reported Outcomes

Self-Reported Outcomes	T1: Baseline Evaluation		
	Experimental (*n* = 20)	Control (*n* = 20)	ANOVA F(*p*)
POMS			
Tension/Anxiety	9.85 (7.39–12.30)	7.00 (3.87–10.12)	2.25 *(0.14)*
Depression/Dejection	12.90 (7.51–18.28)	9.10 (4.44–13.75)	1.24 *(0.27)*
Anger/Hostility	9.50 (5.98–13.01)	8.65 (4.67–12.62)	0.11 *(0.73)*
Vigor/Activity	24.40 (20.23–28.56)	28.75 (25.34–32.15)	2.86 *(0.09)*
Fatigue/Inertia	11.75 (9.51–13.98)	9.20 (7.26–11.13)	3.25 *(0.07)*
Confusion/Bewilderment	8.60 (5.37–11.82)	6.55 (2.91–10.18)	0.77 *(0.38)*
TMD	128.20 (113.47–142.93)	111.75 (96.55–126.95)	2.64 *(0.11)*
EORTC QLQ-C30			
Global health status/QoL	53.74 (41.84–65.64)	57.08 (46.77–67.38)	0.19 *(0.66)*
Functional scales			
Physical functioning	67.61 (56.17–79.04)	76.91 (67.47–86.35)	1.72 *(0.19)*
Role functioning	75.83 (60.75–90.90)	79.16 (64.87–93.45)	0.11 *(0.73)*
Emotional functioning	72.56 (59.55–85,57)	79.68 (69.72–89.63)	0.82 *(0.36)*
Cognitive functioning	81.66 (69.29–94.03)	79.99 (67.96–92.02)	0.04 *(0.84)*
Social functioning	79.99 (64.68–95.30)	90.83 (83.04–98.62)	1.74 *(0.19)*
Symptoms scales			
Fatigue	51.07 (40.48–61.65)	40.53 (30.50–50.56)	2.28 *(0.13)*
Nausea and vomiting	0.87 (0.96–2.71)	1.66 (0.73–4.06)	0.29 *(0.59)*
Pain	50.83 (38.31–63.35)	38.33 (27.27–49.39)	2.45 *(0.12)*
Dyspnoea	6.66 (−4.18–17.52)	8.33 (−2.84–19.50)	0.05 *(0.82)*
Insomnia	53.33 (37.00–69.65)	44.99 (25.87–64.12)	0.48 *(0.49)*
Appetite loss	14.99 (2.12–27.87)	14.99 (3.15–26.84)	0.00 *(1.00)*
Constipation	4.99 (−2.63–12.63)	8.33 (−1.63–18.29)	0.30 *(0.58)*
Diarrhoea	11.66 (0.04–23.28)	6.66 (−2.93–16.28)	0.48 *(0.49)*
Financial difficulties	8.33 (−3.93–20.60)	14.99 (2.12–27.87)	0.61 *(0.43)*
VAS	7.40 (6.82–7.98)	6.65 (5.98–7.32)	3.17 *(0.08)*
SFI	57.40 (49.59–65.20)	56.90 (47.66–66.13)	0.007 *(0.93)*
ULFI	61.80 (53.93–69.66)	69.60 (62.07–77.12)	2.25 *(0.14)*
BAI	0.59 (0.47–0.71)	0.42 (0.28–0.55)	4.07 *(0.05)*
MBS	0.40 (0.23–0.56)	0.33 (0.21–0.44)	0.53 *(0.46)*
BADIX	0.53 (0.41–0.65)	0.40 (0.28–0.51)	2.93 *(0.09)*

*Abbreviations: POMS* Profile of Mood States, *TMD* Total Emotional Distortion, *EORTC QLQ-C30* European Organization for Research and Treatment of Cancer (EORT) Quality of Life Questionnaire QLQ-C30, *QoL* Quality of Life, *VAS* Visual Analogue Scale, *SFI* Spine Functional Index, *ULFI* Upper Limb Functional Index, *BAI* Backache Index, *MBS* Morning Stiffness Score, *BADIX* Backache Disability Index

### Changes in quality of life (EORTC QLQ-C30), profile of mood states POMS, visual analogue scale (VAS) and functional index (SFI-Sp, ULFI-Sp and BADIX)

#### Changes between control and intervention groups

##### Time 1-time 2

In this intergroup analysis we found no differences between the groups a week later the first intervention (Table 3).

**Table 3:** Mean (CI 95%) and analysis of variance (ANOVA) of baseline. 1 and 5 week Patient-Reported Outcomes

Self-Reported Outcomes	T2: 1 week Evaluation			T3: 5 week Evaluation		
	Experimental (n = 20)	Control (n = 20)	ANOVA F(*p*)	Experimental (n = 20)	Control (*n* = 20)	ANOVA F(*p*)
POMS						
Tension/Anxiety	6.05 (3.26–8.83)	6.50 (3.52–9.47)	0.05 *(0.81)*	5.40 (2.77–8.02)	6.50 (3.89–9.10)	0.38 *(0.53)*
Depression/Dejection	8.00 (3.76–12.23)	10.05 (5.26–14.83)	0.45 *(0.50)*	6.35 (2.71–9.98)	9.30 (4.41–14.19)	1.02 *(0.31)*
Anger/Hostility	5.40 (3.20–7.59)	5.75 (2.51–8.98)	0.03 *(0.85)*	4.25 (2.51–5.98)	6.60 (2.69–10.50)	1.32 *(0.25)*
Vigor/Activity	24.80 (20.46–29.13)	26.70 (23.13–30.26)	0.50 *(0.48)*	28.10 (24.09–32.10)	25.90 (21.85–29.94)	0.65 *(0.42)*
Fatigue/Inertia	8.85 (6.49–11.20)	8.75 (6.56–10.93)	0.004 *(0.94)*	6.40 (4.33–8.46)	8.00 (5.76–10.23)	1.21 *(0.27)*
Confusion/Bewilderment	5.95 (3.10–8.79)	7.10 (3.52–10.67)	0.27 *(0.60)*	3.75 (0.84–6.65)	5.00 (1.55–8.44)	0.33 *(0.56)*
TMD	109.90 (97.14–122.66)	111. 30 (95.33–127.27)	0.02 *(0.88)*	98.05 (85.33–110.77)	109.40 (93.87–124.93)	1.40 *(0.24)*
EORTC QLQ-C30						
Global health status/QoL	67.49 (59.50–75.48)	63.74 (56.55–70.93)	0.53 *(0.47)*	76.66 (69.66–83.66)	62.91 (56.15–69.66)	8.75 *(0.005)*
Functional scales						
Physical functioning	78.68 (69.12–88.24)	74.03 (62.34–85.71)	0.41 *(0.52)*	80.18 (69.45–90.90)	75.03 (63.70–86.35)	0.47 *(0.49)*
Role functioning	85.83 (73.88–97.77)	70.83 (54.85–86.81)	2.47 *(0.12)*	91.66 (82.71–100.61)	79.16 (68.76–89.56)	3.63 *(0.06)*
Emotional functioning	83.78 (75.36–92.20)	77.96 (65.83–90.09)	0.68 *(0.41)*	85.90 (78.35–93.45)	81.36 (71.89–90.83)	0.61 *(0.43)*
Cognitive functioning	91.76 (82.02–101.30)	84.99 (73.42–96.56)	0.85 *(0.36)*	92.49 (84.70–100.28)	84.16 (72.71–95.61)	1.58 *(0.21)*
Social functioning	87.49 (74.87–100.12)	86.66 (75.46–97.86)	0.01 *(0.91)*	91.66 (81.70–101.63)	94.99 (89.28–100.71)	0.36 *(0.54)*
Symptoms scales						
Fatigue	26.62 (18.62–34.61)	37.76 (28.16–47.37)	3.48 *(0.07)*	21.62 (12.46–30.78)	39.42 (28.55–50.30)	6.87 *(0.01)*
Nausea and vomiting	0.00 (0.00–0.00)	0.83 (−0.91–2.57)	1.00 *(0.32)*	1.66 (− 0.73–4.06)	0.00 (0.00–0.00)	2.11 *(0.15)*
Pain	29.16 (17.88–40.44)	31.66 (20.37–42.95)	0.10 *(0.74)*	19.99 (10.00–29.94)	34.16 (24.20–44.11)	4.41 *(0.04)*
Dyspnoea	5.00 (−5.46–15.46)	8.33 (− 2.84–19.50)	0.20 *(0.65)*	3.33 (− 3.64–10.30)	4.99 (− 2.63–12.63)	0.11 *(0.73)*
Insomnia	29.99 (14.89–45.09)	29.99 (14.89–45.09)	0.00 *(1.00)*	23.33 (9.84–36.81)	23.33 (8.92–37.73)	0.00 *(1.00)*
Appetite loss	11.66 (1.20–22.12)	18.33 (1.95–34.71)	0.51 *(0.47)*	11.66 (1.20–22.12)	14.99 (3.15–26.84)	0.19 *(0.66)*
Constipation	1.66 (−1.82–5.15)	9.99 (−0.24–20.24)	2.59 *(0.11)*	1.66 (−1.82–5.15)	6.66 (−2.93–16.26)	1.04 *(0.31)*
Diarrhoea	8.33 (−0.24–16.91)	4.99 (−2.63–12.63)	0.36 *(0.54)*	3.33 (− 1.46–8.13)	8.33 (− 0.24–16.91)	1.13 *(0.29)*
Financial difficulties	3.33 (3.64–10.30)	8.33 (− 1.63–18.29)	0.74 *(0.39)*	3.33 (− 3.64–10.30)	4.99 (− 2.63–12.63)	0.11 *(0.73)*
VAS	6.10 (5.44–6.76)	6.15 (5.26–7.04)	0.009 *(0.92)*	4.90 (4.03–5.77)	6.45 (5.65–7.25)	7.56 *(0.009)*
SFI	67.20 (60.36–74.03)	59.80 (53.77–65.82)	2.88 *(0.09)*	66.80 (57.08–76.51)	59.20 (53.41–64.98)	1.97 *(0.16)*
ULFI	72.60 (65.38–79.81)	72.20 (67.36–77.03)	0.009 *(0.92)*	72.40 (62.64–82.15)	70.60 (64.36–76.83)	0.10 *(0.74)*
BAI	0.42 (0.32–0.51)	0.44 (0.31–0.57)	0.11 *(0.73)*	0.33 (0.24–0.43)	0.40 (0.27–0.56)	0.83 *(0.36)*
MBS	0.19 (0.59–0.32)	0.33 (0.19–0.46)	2.46 *(0.12)*	0.17 (0.05–0.28)	0.33 (0.18–0.47)	3.23 *(0.08)*
BADIX	0.35 (0.25–0.45)	0.42 (0.30–0.54)	0.84 *(0.36)*	0.28 (0.19–0.37)	0.39 (0.26–0.51)	2.16 *(0.14)*

*Abbreviations: POMS* Profile of Mood States, *TMD* Total Emotional Distortion, *EORTC QLQ-C30* European Organization for Research and Treatment of Cancer (EORT) Quality of Life Questionnaire QLQ-C30, *QoL* Quality of Life, *VAS* Visual Analogue Scale, *SFI* Spine Functional Index, *ULFI* Upper Limb Functional Index, *BAI* Backache Index, *MBS* Morning Stiffness Score, *BADIX* Backache Disability Index

##### Time 1-time 3

Observing the values of ANOVA in T3 (at 5 weeks of the start of the study, after the second intervention), we found significant differences favouring the experimental group with respect to variables related to QoL, as Global health status / QoL (*p* = 0.005), fatigue (*p* = 0.01) and pain (*p* = 0.04)). Moreover we found significative differences on the visual analogue scale (VAS) (*p* = 0.009). On the other hand, we did not found differneces related to Profile of Moods State (POMS) neither functional index (SFI, ULFI, BAI, MBS o BADIX).

#### Changes within experimental group

##### Time 1-time 2

When analyzing intra-group variations from the baseline (T1) to the first intervention (T2), in the experimental group we observed that intra-group changes occur early, after the first intervention, in variables such as the state questionnaire (POMS), except for the vigor-activity variable. Intra-group changes occur after the first intervention in several variables relating to the quality of life such as global health status (*p* = 0.002), several functional scales (role (*p* = 0.03), emotional (*p* = 0.008)), symptoms such as fatigue (*p* = 0), pain (*p* = 0) and insomnia (*p* = 0.009). Significatibe differences were observed in the VAS punctuation (*p* = 0) and at different functional indexes: the spine (*p* = 0), the upper limb (*p* = 0) and those that value disability backache (BADIX (*p* = 0), BAI (0.001) and MBS (0.003)) (Table 4).

**Table 4:** Mean and Range T_1_ (Baseline). T_2_ (1 week after) and T_3_ (5 week after); Mean Differences. t-distribution. and *p* values of T_1_ - T_2_ and T_1_- T_3_ for within-group change scores in Patient-Reported Outcomes

Self-Reported Outcomes	Experimental							Control						
	T_1_	T_2_	T_3_	Mean differences T_1_ - T_2_	t (*p*) value T_1_ - T_2_	Mean differences T_1_ - T_3_	t (*p*) value T_1_ - T_3_	T_1_	T_2_	T_3_	Mean differences T_1_ - T_2_	t (*p*) value T_1_ - T_2_	Mean differences T_1_ - T_3_	t (*p*) value T_1_ - T_3_
POMS														
Tension/Anxiety	9.85 (0–17)	6.05 (−4–17)	5.40 (−4–17)	3.80	3.98 *(0.001)*	4.45	4.45 *(0.00)*	7.00 (−2–24)	6.5 (−3–20)	6.5 (− 2–19)	0.50	0.45 *(0.65)*	0.5	0.63 *(0.53)*
Depression/Dejection	12.90 (0–40)	8 (0–31)	6.35 (0–24)	4.90	3.54 *(0.002)*	6.55	3.90 *(0.001)*	9.10 (0–36)	10.05 (0–33)	9.3 (0–33)	−0.95	−0.62 *(0.54)*	− 0.2	− 0.13 *(0.89)*
Anger/Hostility	9.50 (0–26)	5.40 (0–15)	4.25 (1–12)	4.10	3.2 *(0.005)*	5.25	3.63 *(0.002)*	8.65 (0–25)	5.75 (0–28)	6.6 (0–28)	2.90	2.05 *(0.05)*	2.05	1.54 *(0.13)*
Vigor/Activity	24.40 (10–42)	24.80 (9–43)	28.10 (10–43)	−0.4	−0.28 *(0.78)*	−3.70	−2.98 *(0.008)*	28.75 (17–43)	26.70 (11–40)	25.9 (11–40)	2.05	1.91 *(0.07)*	2.85	2.18 *(0.04)*
Fatigue/Inertia	11.75 (2–21)	8.85 (0–21)	6.40 (0–15)	2.90	2.86 *(0.01)*	5.35	*4.42 (0.00)*	9.20 (2–16)	8.75 (2–16)	8.00 (0–18)	0.45	0.56 *(0.57)*	1.20	1.45 *(0.16)*
Confusion/Bewilderment	8.6 (0–23)	5.95 (−2–19)	3.75 (−3–17)	2.65	2.80 *(0.01)*	4.85	4.50 *(0.00)*	6.55 (−3–24)	7.10 (−2–24)	5.00 (− 2–24)	− 0.55	− 0.72 *(0.47)*	1.55	1.38 *(0.18)*
TMD	128.20 (84–189)	109.90 (80–166)	98.05 (68–153)	18.30	3.45 *(0.003)*	30.15	*5.97 (0.00)*	111.75 (67–193)	111.30 (68–194)	109.4 (63–194)	0.45	0.11 *(0.90)*	2.35	0.53 *(0.59)*
EORTC QLQ-C30														
Global health status/QoL	53.76 (0–83.33)	67.49 (4–83.33)	76.66 (50–100)	−13.75	−3.62 *(0.002)*	−22.91	−4.18 (0.00)	57.08 (0–83.33)	63.74 (33.33–91.66)	62.91 (33.33–83.33)	−6.66	− 1.39 *(0.18)*	− 5.83	− 1.16 *(0.25)*
Functional scales														
Physical functioning	67.61 (0–100)	78.68 (27–100)	80.18 (20–100)	−11.06	−2.41 *(0.26)*	− 12.56	− 2.75 (0.01)	76.91 (33.33–100)	74.03 (27–100)	75.03 (27–100)	2.88	0.96 *(0.34)*	1.88	0.82 *(0.41)*
Role functioning	75.83 (0–100)	85.83 (0–100)	91.66 (33.33–100)	−10	−2.34 *(0.03)*	− 15.83	−3.22 (0.004)	79.16 (0–100)	70.83 (0–100)	79.16 (33–100)	8.33	1.20 *(0.24)*	0.001	0.00 (*1.00)*
Emotional functioning	72.56 (16.66–100)	83.78 (50–100)	85.90 (50–100)	−11.21	−2.95 *(0.008)*	−13.33	−2.53 (0.02)	79.68 (25–100)	77.96 (25–100)	81.36 (33.33–100)	1.71	*0.39 (0.69)*	−1.68	−0.38 *(0.70)*
Cognitive functioning	81.66 (16.66–100)	91.66 (16.66–100)	92.49 (33.33–100)	−10	−1.98 *(0.62)*	− 10.83	−2.66 (0.01)	79.99 (16.66–100)	84.99 (0–100)	84.16 (16.66–100)	−5.00	−1.37 *(0.18)*	−4.16	− 0.72 (*0.48)*
Social functioning	79.99 (0–100)	87.49 (0–100)	91.66 (33.33–100)	−7.50	−1.69 *(0.10)*	− 11.66	−2.40 (0.02)	90.83 (50–100)	86.66 (0–100)	94.99 (66.66–100)	4.16	0.62 *(0.54)*	−4.16	*−0.81 (0.42)*
Symptoms scales														
Fatigue	51.07 (11–88.66)	26.62 (11–77.66)	21.62 (0–77.66)	24.45	5.66 *(0.00)*	29.44	7.25 (0.00)	40.53 (0–77.77)	37.76 (0–77.77)	39.42 (0–77.77)	2.76	*0.75 (0.46)*	1.10	0.26 *(0.79)*
Nausea and vomiting	0.87 (0–16.66)	0.00	1.75 (0–16.66)	0.87	1.00 *(0.33)*	−0.87	−0.56 (0.57)	1.66 (0–16.66)	0.83 (0–16.66)	0.00	0.83	1.00 *(0.33)*	1.66	1.45 *(0.16)*
Pain	50.83 (0–100)	29.16 (0–83.33)	19.99 (066.66)	21.66	4.61 *(0.00)*	30.83	5.80 (0.00)	38.33 (0–83.33)	31.66 (0–66.66)	34.16 (0–66.66)	6.66	1.32 *(0.20)*	4.16	*1.04 (0.30)*
Dyspnoea	6.66 (0–100)	5.00 (0–100)	3.33 (0–66.66)	1.66	1.00 *(0.33)*	3.33	1.45 (0.16)	8.33 (0–100)	8.33 (0–100)	4.99 (0–66.66)	0.00	0.00 *(1.00)*	3.33	*1.00 (0.33)*
Insomnia	53.33 (0–100)	29.99 (0–100)	23.33 (0–66.66)	23.33	2.89 *(0.009)*	29.99	4.41 (0.00)	44.99 (0–100)	29.99 (0–100)	23.33 (0–100)	15	2.43 *(0.02)*	21.66	3.32 *(0.004)*
Appetite loss	14.99 (0–100)	11.66 (0–66.66)	11.66 (0–66.66)	3.33	*0.69 (0.49)*	3.33	0.81 (0.42)	14.99 (0–66.66)	18.33 (0–100)	14.99 (0–66.66)	−3.33	−0.49 *(0.62)*	0.00	0.00 *(1.00)*
Constipation	4.99 (0–66.66)	1.66 (0–33.33)	1.66 (0–33.33)	3.33	1.00 *(0.33)*	3.33	0.80 (0.42)	8.33 (0–66.66)	9.99 (0–66.66)	6.66 (0–66.66)	−1.66	*−0.32 (0.74)*	1.66	0.27 *(0.78)*
Diarrhoea	11.66 (0–66.66)	8.33 (0–66.66)	3.33 (0–33.33)	3.33	0.62 *(0.54)*	8.33	1.75 (0.09)	6.66 (0–66.66)	4.99 (0–66.66)	8.33 (0–66.66)	1.66	0.43 *(0.66)*	−1.66	−0.37 *(0.71)*
Financial difficulties	8.33 (0–100)	3.33 (0–66.66)	3.33 (0–66.66)	5.00	1.00 *(0.33)*	5.00	1.00 (0.33)	14.99 (0–66.66)	8.33 (0–66.66)	4.99 (0–66.66)	6.66	1.45 *(0.16)*	9.99	1.83 *(0.08)*
VAS	7.4 (5–10)	6.1 (4–9)	4.90 (1–8)	1.30	*7.93 (0.00)*	*2.50*	8.01 *(0.00)*	6.65 (4–9)	6.15 (2–9)	6.45 (3–9)	0.50	2.12 *(0.47)*	0.20	1.16 *(0.25)*
SFI	57.40 (8–84)	67.20 (20–84)	66.80 (0–92)	−9.8	−5.24 *(0.00)*	−9.40	−1.74 *(0.09)*	56.90 (6–96)	59.80 (44–88)	59.20 (40–84)	−2.90	*−0.93 (0.36)*	−2.30	−0.65 *(0.51)*
ULFI	61.80 (16–84)	72.60 (28–92)	72.40 (0–92)	−10.80	−6.89 *(0.00)*	−10.60	− 2.05 *(0.05)*	69.60 (40–100)	72.20 (48–100)	70.60 (48–96)	−2.60	−0.95 *(0.35)*	−1.00	−0.26 *(0.79)*
BAI	0.59 (0.07–1.00)	0.42 (0.13–0.93)	0.33 (0.07–0.93)	0.17	4.01 *(0.001)*	0.25	4.52 (*0.00)*	0.42 (0–0.86)	0.44 (0–0.80)	0.40 (0–0.80)	−0.02	*−0.90 (0.37)*	0.01	0.44 *(0.66)*
MBS	0.40 (0.00–1.00)	0.19 (0–0.80)	0.17 (0–0.80)	0.21	3.46 *(0.003)*	0.23	3.61 *(0.002)*	0.33 (0–0.80)	0.33 (0–0.80)	0.33 (0–0.80)	0.00	0.00 *(1.00)*	0.00	0.00 *(1.00)*
BADIX	0.53 (0.02–1.00)	0.35 (0.01–0.90)	0.28 (0.02–0.90)	0.18	4.80 *(0.00)*	0.25	5.59 *(0.00)*	0.40 (0–0.80)	0.42 (0–0.75)	0.39 (0–0.75)	−0.02	−0.93 *(0.36)*	0.01	0.49 *(0.62)*

*Abbreviations: POMS* Profile of Mood States, *TMD* Total Emotional Distortion, *EORTC QLQ-C30* European Organization for Research and Treatment of Cancer (EORT) Quality of Life Questionnaire QLQ-C30, *QoL* Quality of Life, *VAS* Visual Analogue Scale, *SFI* Spine Functional Index, *ULFI* Upper Limb Functional Index, *BAI* Backache Index, *MBS* Morning Stiffness Score, *BADIX* Backache Disability Index

##### Time 1-time 3

When analyzing intra-group variations from the baseline (T1) to the second intervention (T3), in the experimental group, we found significant differences in all aspects of the POMS, except for the variable vigor/activity. These statistically significant changes also occur in most of the variables evaluated in the EORTC QLQ-C30 quality of life questionnaire. Statistically significant changes were observed in the overall health status (*p* = 0.01); Physical functioning (*p* = 0.01), role (*p* = 0.004), emotional (*p* = 0.02), cognitive (*p* = 0.01) and social (*p* = 0.02); We also observed differences in symptoms such as fatigue (*p* = 0), pain, (*p* = 0) insomnia (*p* = 0) and changes in the EVA scale (*p* = 0). Changes were observed in almost all functional indexes, such as ULFI (*p* = 0.05), BAI (p = 0), MBS (*p* = 0.002) and BADIX (*p* = 0) (Table 4).

#### Changes within control group

##### Time 1-time 2

When analyzing intra-group variations from the baseline (T1) to the first intervention (T2), in the control group we observed that only significant differences were observed in the anger/hostility variable of the POMS scale (p = 0.05), and In the insomnia variable of the EORTC quality of life questionnaire (*p* = 0.02) (Table 4).

##### Time 1-time 3

When analyzing the intra-group variations from the baseline (T1) to the second intervention (T3), in the control group we observed that the benefit in the anger / hostility variable was not maintained until the end of the study (*p* = 0.13), However, there were significant differences at the end of the intervention in the varibale vigor-activity (POMS scale) (*p* = 0.04), as well as in the insomnia variable of the EORTC quality of life questionnaire (*p* = 0.004) (Table 4).

### Changes in the dynamometry and pressure pain threshold

#### Changes between control and intervention groups

Table 5 analyze the objective variables inter-group changes over 6 times in which we recorded these variables. The values of the hand grip strength have improved in the experimental group, both affects and healthy hand. Despite this strength gain that can be seen in the improvement of the means, the change is not statistically significant (healthy hand *p* = 0.54; affected hand *p* = 0.77). In the control group hardly seen changes in the grip strength of the participants. Therefore, we can affirm that there were no statistically significant inter-group differences with respect to dynamometry, nor were differences observed with respect to the pain threshold.

**Table 5:** Change in grip strength (kg) and pressure pain threshold from baseline to 5 week neuromuscular taping therapy

Measure	Groups			
	Experimental		Control	
	Mean (CI 95%)	F(*p*)	Mean (CI 95%)	F(*p*)
HG Healthy Hand		0.80 (*0.54*)		0.11 (*0.98*)
Basal (T_1_)	16.45 (13.68–19.21)		18.70 (16.61–20.78)	
After 1st NMT (T_1_)	16.70 (14.13–19.26)		18.32 (16.35–20.29)	
Arrival 1st week (T_2_)	17.31 (14.57–20.05)		17.90 (15.82–19.98)	
After 2nd NMT (T_2_)	17.65 (15.12–20.17)		18.20 (16.09–20.30)	
Arrival 5nd week (T_3_)	19.07 (16.26–21.88)		18.80 (16.79–20.80)	
After 3rd NMT (T_3_)	19.12 (16.44–21.80)		18.45 (16.34–20.55)	
HG Affected Hand		0.49 (*0.77*)		0.18 (*0.96*)
Basal (T_1_)	14.16 (10.99–17.33)		16.85 (14.58–19.11)	
After 1st NMT (T_1_)	13.88 (11.24–16.53)		16.30 (14.09–18.50)	
Arrival 1st week (T_2_)	15.18 (12.43–17.93)		16.65 (14.35–18.94)	
After 2nd NMT (T_2_)	15.12 (12.55–17.69)		16.27 (14.03–18.51)	
Arrival 5nd week (T_3_)	15.87 (13.23–18.51)		17.50 (15.24–19.75)	
After 3rd NMT (T_3_)	16.27 (13.50–19.04)		16.57 (14.50–18.64)	
Cervical PPT		0.35 (*0.88*)		0.44 (*0.81*)
Basal (T_1_)	11.96 (10.12–13.80)		12.31 (10.63–13.99)	
After 1st NMT (T_1_)	12.37 (10.50–14.23)		11.80 (10.13–13.46)	
Arrival 1st week (T_2_)	10.93 (9.24–12.62)		12.08 (10.52–13.63)	
After 2nd NMT (T_2_)	11.35 (9.69–13.01)		11.07 (9.47–12.66)	
Arrival 5nd week (T_3_)	11.73 (9.74–13.71)		11.47 (10.05–12.88)	
After 3rd NMT (T_3_)	11.34 (9.56–13.12)		11.13 (9.55–12.71)	
Lumbar PPT		0.08 (*0.99*)		0.23 (*0.94*)
Basal (T_1_)	13.76 (11.52–16.00)		15.43 (13.35–17.50)	
After 1st NMT (T_1_)	14.38 (12.20–16.56)		14.50 (12.63–16.37)	
Arrival 1st week (T_2_)	14.05 (12.06–16.04)		15.06 (12.61–17.51)	
After 2nd NMT (T_2_)	14.49 (12.46–16.52)		14.25 (12.04–16.45)	
Arrival 5nd week (T_3_)	14.05 (12.07–16.02)		15.35 (13.54–17.16)	
After 3rd NMT (T_3_)	14.45 (12.56–16.34)		15.12 (13.25–16.99)	
Median Nerve PPT		0.46 (*0.80*)		0.42 (*0.83*)
Basal (T_1_)	18.74 (16.27–21.21)		17.99 (15.97–20.01)	
After 1st NMT (T_1_)	18.45 (16.02–20.87)		17.19 (15.34–19.04)	
Arrival 1st week (T_2_)	17.53 (15.76–19.29)		17.51 (15.73–19.28)	
After 2nd NMT (T_2_)	17.43 (15.53–19.32)		16.88 (14.98–18.77)	
Arrival 5nd week (T_3_)	17.16 (15.19–19.13)		16.76 (14.84–18.68)	
After 3rd NMT (T_3_)	17.14 (15.17–19.10)		16.32 (14.43–18.21)	
Tibialis Anterior PPT		0.22 (*0.95*)		0.80 (*0.55*)
Basal (T_1_)	15.39 (13.41–17.37)		17.29 (15.49–19.08)	
After 1st NMT (T_1_)	15.51 (13.53–17.48)		17.10 (15.35–18.84)	
Arrival 1st week (T_2_)	15.02 (13.14–16.89)		16.65 (14.87–18.43)	
After 2nd NMT (T_2_)	14.86 (12.89–16.82)		16.16 (14.17–18.14)	
Arrival 5nd week (T_3_)	14.56 (12.74–16.38)		15.71 (14.04–17.38)	
After 3rd NMT (T_3_)	14.46 (12.70–16.21)		15.39 (13.69–17.08)	

*Abbreviations: HG* Hand Grip, *PPT* Pressure Pain Threshold, *NMT* Neuromuscular Taping. T_1_ (Baseline). T_2_ (1 week after) and T_3_ (5 week after)

**Fig. 3:**
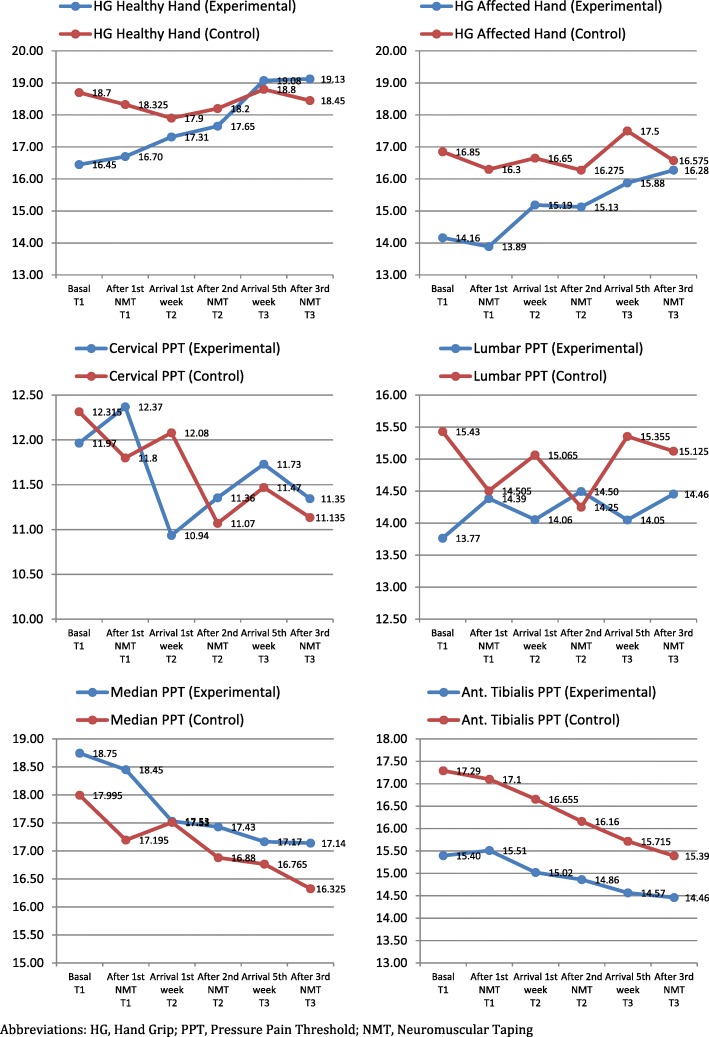
Means of objective measures from both groups at different times of the study

The Fig. 3 represent graphically, changes in objective variables, based on the data contained in Table 5. We observe how the hand strength improvement in the experimental group in the healthy hand and on the side of surgery (albeit with lower values). Instead, records in the group which received the sham intervention, remain within the same range (means of ±18 in healthy side and ± 16 in the affected). In the representation of the cervical and lumbar PPT it tells us how after each application of NMT, (both real and sham) had a slight increase in the threshold values. That is, all participants were less painful sensitivity to pressure after each intervention at these levels. The opposite effect appears to occur when we apply the bandage at the level of the median nerve. After each application the participant, in both groups, support less pressure before the painful sensation appears. In the case of the algometer application on the tibialis anterior of all participants, the results hardly alter pre and post intervention. If we make an overall assessment at this point, the threshold decreases discretely in both groups between baseline and the end, especially in the control group, although as in the previous cases, without statistical significance.

### Changes in the plasmatic proteins

#### Changes between control and intervention groups

When analyzing the variations between the two groups from the baseline (T1) to the second intervention (T3), there were no statistically significant differences in C-reactive protein (CRP) or Creatin-kinase (CK) values (*p* = 0.53 and *p* = 0.99 respectively); So we can say that the intervention has failed to modify these biochemical parameters (Table 6).

**Table 6:** Change scores between-group and within-group in biological outcomes

		Mean (SD)	Mean difference	CI 95%	t (*p*)	
CRP 5th week	Experimental	4.09 (6.51)	−2.48	−10.54 to 5.57	−0.63 (*0.53*)	Between group
	Control	6.57 (11.52)				
CK 5th week	Experimental	105.36 (44.17)	−0.20	−37.92 to 37.50	−0.01 (*0.99*)	
	Control	105.57 (46.05)				
		Mean (SD)	Mean difference	CI 95%	t (*p*)	Within group
Experimental	Basal CRP	4.27 (5.81)	0.182	−0.47 to 0.84	0.61 (*0.55*)	
	5th week CRP	4.09 (6.51)				
Control	Basal CRP	5.36 (5.13)	−1.21	−7.28 to 4.85	−0.43 (*0.67*)	
	5th week CRP	6.57 (11.52)				
Experimental	Basal CK	102.36 (39.08)	−3.00	−16.17 to 10.17	−0.50 (*0.62*)	
	5th week CK	105.36 (44.17)				
Control	Basal CK	112.50 (43.57)	6.92	−12.19 to 26.05	0.78 (*0.44*)	
	5th week CK	105.57 (46.05)				

*Abbreviations: CRP* C-reactive Protein, *CK* Creatine Kinase, *SD* standard deviation, *CI* confidence interval

#### Changes within-groups

Intra-group levels of PCR and CK have not been statistically different before and after the intervention; Both in the experimental group and in the placebo group (Table 6).

## Discussion

Mialgias and arthralgias are increasingly recognised to be a toxicity induced by AI therapy, but the mechanism underlying it development and it management remains unclear. As a result, there is no scientifically supported treatment indicated to treat these symptoms. In this pilot study we try to improve the participants subjective feeling of pain through an intervention using NMT.

After performing this pragmatic clinical trial, we can conclude that in BC patients treated with adjuvant AI, the application of a VNM improves the subjective sensation of pain according to the EVA scale (*p* = 0.009), confirming our initial hypothesis that VNM Could improve the sensation of pain in BC patients with musculoskeletal alterations secondary to the use of IAs. Additionally in our study the application of this therapy improves the subjective perception of their overall health, fatigue and subjective pain sensation according to the EORTC quality of life scale.

Results of this pilot study are consistent with those of Imperatory et al., who investigated the safety and efficacy of NMT in reducing postoperative chest pain after lobectomy for lung cancer. After tape application, VNM patients reported overall less thoracic pain than the control group, assessed by VAS [50]. In contrast another study with BCS taking AIs performed an intervention assessing the effect of acupuncture on reducing musculoskeletal symptoms and they did not observe a statistically significant difference in VAS reduction (*p* = 0.31) between cases and controls [51]. This improvement in VAS besides decreasing the adverse effects of the drugs can influence the treatment adherence. Worsening in VAS is associated with increased risk for early AIs discontinuation [52]. NMV has also been used to improve pain in other groups of patients. In patients with knee osteoarthritis, VNM application demonstrated large decrease in VAS, although unlike our study, from the initial taping application [53]. Instead, in nonspecific low back patients, the same effects was achieved on pain scale cases and controls [54].

There is a significant association between pain and lower health-related quality of life scores in the physical and mental component summary scores [55], so it is not surprising that patients in addition to changing VAS improved their overall health status and symptoms contained in their quality of life questionnaire, such as pain or fatigue. A home-based exercise program provided similar benefits to BCS undergoing AI treatment by reducing joint pain and improving QoL [56]. This study is in line with another that intervened through aerobic exercise and stretching [57]. Galantino et al. analyzed the utility of yoga in these BCS using qualitative methods and concluded that this technique improved QoL and pain, although the sample was very small [58]. However, another pilot study did not achieve changes in pain severity or QoL by an intervention using electroacupuncture [59]. Reducing fatigue could enhance therapeutic outcomes by increasing adherence and is also important following completion of cancer treatment, to get the resumption of pre-cancer lifestyles [60]. Regarding the improvement of fatigue, large number of research studies support the ability of exercise training to alleviate cancer-related fatigue, various meta-analysis revealed that it has a favorable effect when compared to conventional care [60, 61]. Other interventions that reduced fatigue in BCS used Mindfulness-Based Music Therapy [62] and yoga [63]. In contrast to our pilot study, NMV did not reduce fatigue in muscular affections with healthy subjects [64].

It would be very useful to be able to identify early changes in Patient-Reported Outcomes that ultimately stopped treatment as a result of toxicity. Identification of these changes could be used to target interventions in patients at high risk for early discontinuation [52].

Regarding the objective variables, there is a clear trend of improvement in grip force of the healthy and affects hand after the intervention, although not statistically significant. In order to evaluate these results, it is necessary to take into account that this variable does not remain stable during the administration of the AIs, grip strength in the left and right hands decrease after 6 months of antihormonal therapy [13]. This gain is hardly seen between controls. Consistent with our findings, women in a resistance and impact training program significantly improved their grip strength (*p* < 0.01) compared to a stretching placebo program [65]. In contrast to this trial, (*p* = 0.47) a group of participants who performed an aerobic exercise and supervised strength training during 12 months, showed no improvement in their grip strength [57].

BC may increase the pain experience at distant sites presumably via alterations in neuroendocrine profiles or direct sensitizing effects on the Central Nervous System [42]. Besides this painful sensitization it is greater if the patient is practiced mastectomy instead of a lumpectomy [66]. In fact, in previous studies, the presence of central nervous system hypersensitivity has already been reported in BCS [67]. The results of this pilot study indicate that the subject’s pressure sensitivity does not change significantly at any level or group after taping applications. Only a slight increase of its values in the cervical and lumbar zone was obtained. Similarly, previous findings suggest that an intervention through myofascial release technique also fails to change the PPT values in BCS over the cervical spine [68]. This may be because three interventions may not be sufficient to activate pain inhibitory mechanisms. Our results are in line with Henry y cols trial, where no statistically significant change in PPT was identified following estrogen deprivation [69]. Taking into account these new findings, it is possible that our intervention did not achieve changes because estrogen depletion have not effects on pain sensibility.

The coexistence of arthralgia, fatigue, and insomnia is associated with elevated C-reactive protein and other inflammatory biomarkers. This suggest a possible inflammatory mechanism underlying these common symptoms [38]. Instead, there are a negative correlation between serum CK levels and BC stage. Its level, which may reflect the status of host immunity, may be an important factor in determining BC development and progression. The results of our trial indicating that plasma proteins such as CRP and CK exhibiting no significant changes after the intervention, at the time of the last assessment, between cases and controls. Besides, there were no differences between the beginning and the end of the trial or in cases or controls for these variables. These results are in line with another study with BCS which present lymphedema, each experimental session involved standard resistance exercises and there were no significant changes in CRP or the CK at the end of the intervention [70]. A similar, non-significant trend was observed for the CRP with a 12-week yoga intervention in fatigued BCS [63]. No changes is observed when intervention, in this case a 12-month moderate-intensity physical activity intervention, is performed in elderly men and women [71]. In contrast to our work, other authors such as Rock et al. decreased blood content of CRP changing the diet composition in overweight/obese women [72].

One limitation of this study is the small sample size which we have recruited, since it is a pilot study, and a possible selection bias produced by those who agree to participate in this type of studies given the time requirements. Furthermore, because our study relies on self-report, there is some degree of misclassification bias because of the existence of the patient’s perception; In spite of this, for subjective symptoms like AIA, patient-reported outcome is considered the gold standard. Another limitation of this study is the focus on systemic (observing changes in plasma proteins) instead of local. Others authors have demostrated using magnetic resonance imaging that local inflammatory processes occurs [73]. Therefore, future studies could be designed to investigate changes at the local level.

In spite of these limitations, this study is, to the best of our knowledge, the first study to date evaluating in BCS con AIA, the association of patient-reported symptoms with an intervention by neuromuscular taping. Strengths of our study include the randomized design, and a focus on women experiencing arthralgia resulting from AI use. It was also an advantage to have the high persistence of the subjects to the intervention and the fact that all measures were taken by the same examiner, which improves the reliability. Finally, it is important to note that no significant side effects were associated with NMT treatment.

A future replication study is needed, with a larger subjects sample, to verify the results identified in our trial. It would also be interesting to relate these results to the hormone therapy regimen administered, that is, if they started with tamoxifen before switching to AIs. In addition to analyzing the impact of the taping, we could verify whether this arthralgia is actually more likely in women switching from tamoxifen to an AI compared with women starting with AI [12]. It would also be interesting to include genetic variables in our study and to analyze their possible relationship with AIA and the impact of the intervention on the outcome variables. Although some benefit of taping was observed after 5 weeks of intervention, we will probably get better results if we extend the application over time. It would also be interesting to know how many interventions are optimal to obtain the desired improvement, as well as a posteriori control to verify that the results have been maintained over time.

## Conclusion

In conclusion, we have shown that after 5 week of NMT therapy, patients treated with an AI experience an improvement of preexisting musculoskeletal symptoms, mainly of their subjective sensation of pain. Furthermore, this passive intervention achieved significant changes in variables related to quality of life such as global health, fatigue or pain, with a strong impact on the subject well-being. Considering the effectiveness of AIs preventing BC recurrences and the proportion of women who discontinue this therapy because of AIAA, interventions designed to minimize the adverse effects of these therapies are useful and necessary. An intervention by NMT to MSCM under treatment with AIs improves their subjective sensation of pain. In addition, this taping had an impact on variables related to the quality of life. This pilot study may be the basis for others to support the use of NMT for the treatment of AIAs, thereby improving their well-being and reducing the dropout rate.

## Abbreviations

AIA: AI- induced arthralgia
AIs: Aromatase Inhibitors
ATAC: Arimidex and tamoxifen alone or in combination
BADIX: Backache Disability Index
BAI: Back Pain Index
BC: Breast cancer
BCS: Breast cancer survivors
CK: Creatine Kinase
CRP: C-reactive Protein
CTS: Carpal Tunnel Syndrome
EORTC QLQ - C30: European Organization for Research and Treatment Cancer Quality of Life Questionnaire
HR: Hormone receptor
IES: Intergroup Exemestane Study
MBS: Morning Stiffness in the Back
MS: Mass Spectrometry
NMT: Neuromuscular Taping
NSAIDs: Nonsteroidal anti-inflammatory drugs
POMS: Profile of Mood States
PPT: Pressure Pain Threshold
QoL: Quality of Life
SFI: Spine Functional Index
ULFI: Upper Limb Functional Index
VAS: Visual Analogue Scale
